# CADA-Net: cross-altitude domain-adaptive perception for label-efficient onboard sensing in autonomous UAVs

**DOI:** 10.3389/fnbot.2026.1926034

**Published:** 2026-10-01

**Authors:** Liya Cai

**Affiliations:** School of Intelligent Management, Suzhou Industrial Park Institute of Services Outsourcing, Suzhou, China

**Keywords:** cross-altitude domain adaptation, feature-wise linear modulation, onboard aerial perception, teacher-student self-training, UAV object detection

## Abstract

Autonomous unmanned aerial vehicles (UAVs) depend on onboard perception as the front end of their sensing-decision-control loop, where each detection feeds directly into the navigation, avoidance, and tracking decisions made in flight. Yet detectors trained at one altitude routinely degrade when the platform is redeployed at another, because the underlying distribution shift couples scale, viewpoint, and target density. This study addresses the cross-altitude transfer gap by proposing CADA-Net, an altitude-conditioned detector for onboard UAV perception that treats altitude as an exploitable domain variable rather than a nuisance to be removed. The framework injects a categorical altitude prior into multi-scale features through feature-wise linear modulation, aligns cross-altitude representations via a gradient-reversal adversarial discriminator attached to each pyramid level, and generates target-domain pseudo-supervision through a weak-to-strong teacher-student self-training stream, all sharing a single conditioned encoder. Together these components target not raw recognition accuracy alone but perception that remains stable as the platform’s viewing geometry and object density change through flight. Evaluated on the UAVDT benchmark under a low → high adaptation protocol, CADA-Net reaches 46.3 mAP@0.5 on the high-altitude band, surpassing the source-only baseline by 17.9 points and the strongest domain-adaptive competitor by 7.2 points, while leaving only a 6.3-point gap to a fully-supervised upper bound; with 5% of target labels, accuracy rises to 48.1 mAP@0.5. Onboard profiling on a Jetson AGX Orin sustains 42 FPS at INT8 precision within a 27.4 W envelope, indicating that the accuracy gain survives the throughput and power limits of embedded inference, which is a precondition for onboard use rather than evidence of closed-loop flight. These findings indicate that rendering a geometric prior explicit can support label-efficient perception at embedded inference rates for UAV platforms operating across altitude transitions.

## 1 Introduction

Onboard perception sits at the centre of the sensing, decision, and control loop that supports autonomous unmanned aerial vehicles (UAVs). Reliable detection of vehicles and other ground objects feeds directly into downstream navigation, collision avoidance, and target tracking: a missed small vehicle at high altitude can delay avoidance or produce an unsafe waypoint, whereas false positives can trigger unnecessary detours and unstable replanning. Recent advances in reinforcement learning for motion control (Hu et al., 2023) and in graph-based mission planning for cooperative platforms (Ma et al., 2024) have extended the range of tasks that mission UAVs can carry out, with operational deployments now spanning search and rescue (Lyu et al., 2023), precision agriculture (Guebsi et al., 2024), and large-scale urban monitoring (Xu et al., 2023). Recent UAV control reviews place the same emphasis on perception robustness as a precondition for the broader autonomy stack (Cetinsaya et al., 2024). Recent embodied UAV perception studies in neurorobotics, including NavBLIP for navigation-oriented UAV detection (Li et al., 2025), also frame detection as a functional input to flight decisions rather than as a detached recognition task. A perception backbone that remains stable across altitude transitions therefore matters not merely as an isolated vision module, but as an input to closed-loop decisions made under changing geometry and object density.

The operating envelope of a mission UAV rarely sits at a single altitude. A search platform may sweep a corridor at low altitude before climbing for area coverage, and an inspection drone often transitions between close hovering and high-altitude transit. Each transition shifts target scale, perspective, target density, and effective receptive field in a coupled manner. Detectors trained on imagery captured at one altitude routinely show large drops when redeployed at another, and the underlying distribution shift is geometric and semantic at the same time rather than a pure scale change (Wu et al., 2019; Zhu et al., 2021). Multi-scale and small-object detectors developed on UAV imagery have made steady progress on within-domain scale variation (Jiang et al., 2024; Zhang et al., 2023), as have lightweight transformer pipelines for low-altitude scene parsing (Lu et al., 2024), yet these approaches provide limited safeguard against the cross-altitude transfer gap. Adjacent lines of image analysis converge from different sensing conditions, in that collaborative saliency modelling sharpens the localisation of objects with intricate boundaries in optical remote sensing imagery (Xie et al., 2025), context association separates landslide bodies from visually similar bare ground in aerial photographs (Xie et al., 2024), paired detail and semantic branches stabilise monocular depth estimation in cluttered indoor scenes (Song et al., 2025), and visual attention isolates thin structures from distracting background texture during concrete crack inspection (Song et al., 2026). These studies indicate that an explicit structural or contextual prior outperforms added backbone capacity once the targets are small and the background is heterogeneous, which is the regime that high-altitude UAV imagery presents.

Mainstream domain-adaptive detectors have so far concentrated on weather, illumination, and synthetic-to-real shifts, typically through adversarial feature alignment (Chen et al., 2018; Ganin et al., 2016), self-training with a teacher network (Li et al., 2022; Cao et al., 2023), or hybrids that balance the two signals (Munir et al., 2023). Surveys of unsupervised domain adaptation for detection note that altitude has remained outside the canonical list of factors examined in this literature (Oza et al., 2023). Earlier UAV-specific work treats altitude, viewpoint, and weather as nuisance attributes to be removed (Wu et al., 2019), yet altitude carries an informative geometric prior that can be exploited rather than discarded. Methods that align without conditioning on altitude tend to collapse heterogeneous high-altitude statistics into a single target manifold, which limits transfer once the gap becomes geometric in nature. The cost of fully annotating a target altitude further compounds the problem, since high-altitude imagery in public benchmarks is markedly scarcer than mid- and low-altitude imagery (Yu et al., 2020).

Motivated by these observations, the present study revisits cross-altitude transfer and tests the following hypotheses:

*H1*: Treating altitude as an explicit domain variable, and injecting it into multi-scale features through feature-wise linear modulation, recovers more of the cross-altitude gap than altitude-agnostic alignment.

*H2*: A teacher-student self-training stream coupled with adversarial alignment delivers competitive target-domain accuracy when the target altitude carries few or no annotations.

*H3*: The cross-altitude distribution shift cannot be reduced to a scale-only problem, so a strong multi-scale baseline alone leaves a measurable residual gap that altitude-aware adaptation can narrow.

To examine these hypotheses, the paper develops CADA-Net, a cross-altitude domain-adaptive detector that pairs altitude-conditioned feature modulation with weak-to-strong teacher-student pseudo-labelling. The method is evaluated on the UAVDT benchmark and is profiled on an embedded platform to verify that the gain in target-altitude accuracy does not come at the expense of onboard real-time inference. Beyond its empirical findings, the work positions cross-altitude perception as one component of the broader autonomous UAV stack and offers practical evidence on how a simple geometric prior, rendered explicit, can support label-efficient transfer for embodied platforms.

## 2 Materials and methods

### 2.1 Problem formulation and overall framework of CADA-Net

The cross-altitude transfer problem is formulated as a domain adaptation task in which altitude is treated as an explicit domain variable. Let *D_s_* = {(*X*_s_, *B_s_*, *C_s_*)} denote a source set of low-altitude images with bounding boxes and class labels, and let *D_t_* = {*X_t_*} denote a target set of high-altitude images that are either unlabelled or carry only a small annotation budget. Each image is associated with a categorical altitude tag *h* ∈ {low, medium, high} derived from on-board metadata available in standard UAV benchmarks (Du et al., 2018). The goal is to learn a detector that generalises to the target altitude while drawing supervision predominantly from the labelled source.

CADA-Net retains the two-stage Faster R-CNN backbone (Ren et al., 2015) with a ResNet-101 feature extractor (He et al., 2016) and a feature pyramid neck (Lin et al., 2017). Two parallel learning streams share the same encoder. An altitude-conditioned adversarial alignment stream encourages the pyramid features to be indistinguishable across source and target while remaining modulated by the altitude prior. A weak-to-strong teacher-student self-training stream produces pseudo-supervision on target images that lack annotations. The two streams are coupled indirectly through the shared student encoder, on which the adversarial alignment imposes a persistent drift toward domain-invariant features, and the exponential moving average forwards this slow component to the teacher over many iterations while attenuating the transient fluctuation of individual adversarial updates, which yields a more stable transfer of the aligned representation than purely sequential pipelines (Figure 1).

**Figure 1:**
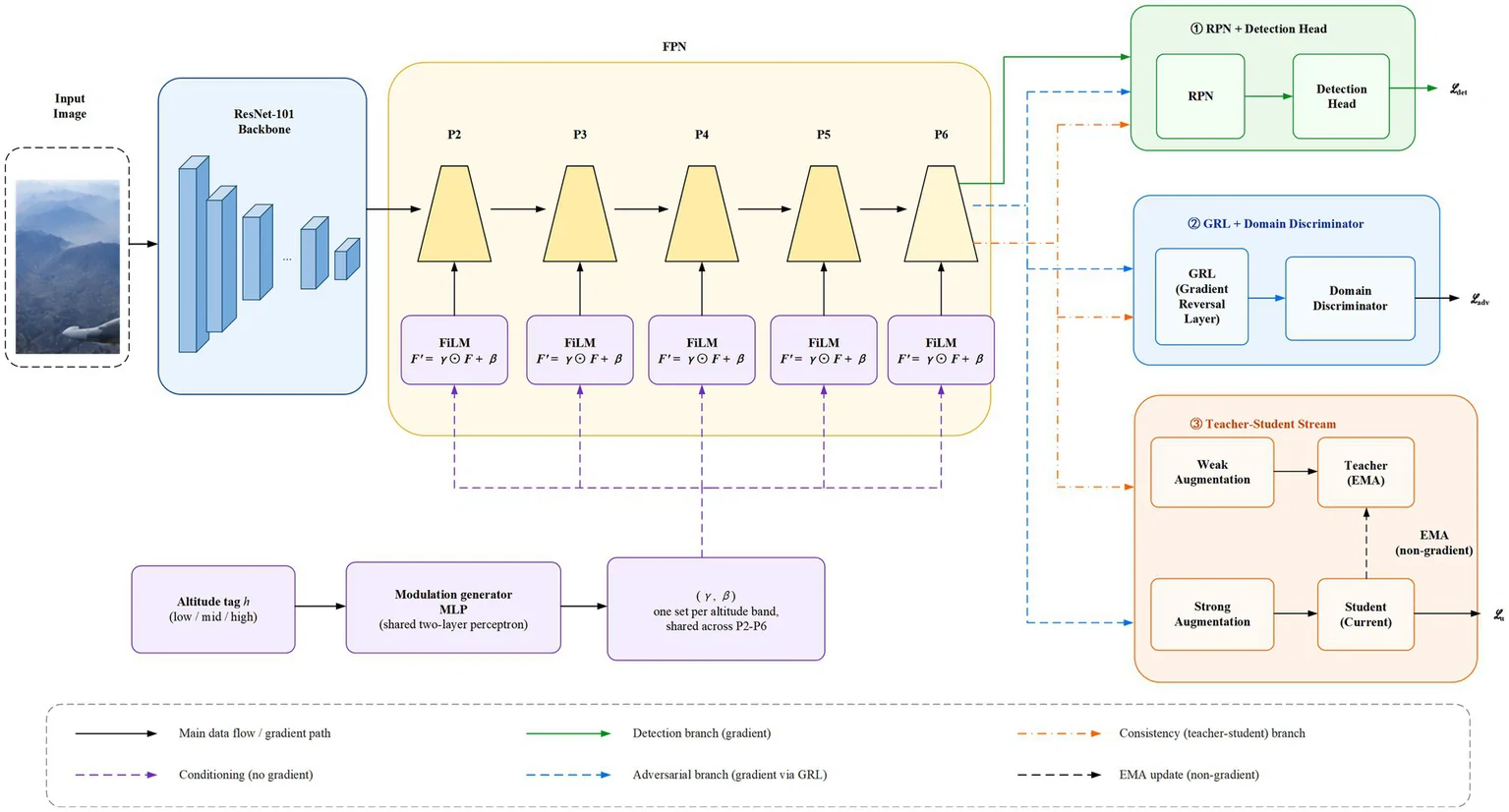
Overall architecture of CADA-Net with the altitude-conditioned FiLM inset.

Three loss terms drive optimisation. A supervised detection loss ${ℒ}_{\det}$ is computed on the labelled source. An adversarial loss ${ℒ}_{\mathrm{adv}}$ , realised through a gradient reversal layer, penalises the encoder for features that the domain discriminator can separate across altitudes. An unsupervised loss ${ℒ}_{u}$ supervises the student on strongly augmented target images using filtered pseudo-boxes produced by the teacher on weakly augmented views. The total objective

$${ℒ}_{\text{total}}={ℒ}_{\det}+\lambda_{\mathrm{adv}}\cdot {ℒ}_{\mathrm{adv}}+\lambda_{u}\cdot {ℒ}_{u}$$ (1)

is jointly minimised, and the altitude prior h is injected into the pyramid features through modulation, as given in Equation 1.

The three terms are defined as follows. The detection loss retains the native two-stage multi-task loss of Faster R-CNN (Equation 2) without modification (Ren et al., 2015),

$${ℒ}_{\det}=\sum_{s\in \{\mathrm{rpn},\text{head}\}}[\frac{1}{N_{\mathrm{cls}}}\sum_{i}{ℒ}_{\mathrm{cls}}(p_{i},p_{i}^{*})+\frac{\lambda }{N_{\mathrm{reg}}}\sum_{i}p_{i}^{*}{ℒ}_{\mathrm{reg}}(t_{i},t_{i}^{*})]$$ (2)

where *pᵢ* and pᵢ^*^ are the predicted and ground-truth labels of anchor or proposal i, *tᵢ* and tᵢ^*^ the predicted and target box-regression offsets, ℒ_cls_ a cross-entropy loss, ℒ_reg_ a smooth-L1 loss evaluated over positive samples only, N_cls_ and N_reg_ the normalisers, and λ the balancing weight. The adversarial loss is a per-location binary cross-entropy averaged over the five pyramid levels (Equation 3) behind the gradient reversal layer,

$$L_{\mathrm{adv}}=-\frac{1}{|P|}\sum_{l\in P}\frac{1}{|\Omega_{l}|}\sum_{u\in \Omega_{l}}[d\log D_{l}(u)+(1-d)\log\;(1-D_{l}(u))]$$ (3)

where *Dₗ*(*u*) is the domain posterior of the shared discriminator at location u of level l, Ωₗ the spatial grid of level l, *P* the level set P2–P6, and d ∈ {0,1} the source-or-target label. The unsupervised loss applies the detection loss to the strongly augmented target view (Equation 4) *x*_t_^s^ using the teacher’s filtered pseudo-boxes B̂ as targets,

$${ℒ}_{u}={ℒ}_{\det}\;(x_{t}^{s},\hat{B})$$ (4)

### 2.2 Altitude-conditioned adversarial feature alignment

The first stream addresses a behaviour that has often been treated implicitly in prior cross-domain detectors. Domain discriminators that map all target samples to a single statistic tend to underuse the structure of the target domain when the shift is geometric. Adversarial alignment under a gradient reversal layer remains effective for appearance shifts such as weather or style (Ganin et al., 2016), yet these formulations do not exploit a directly available geometric cue when one exists. Altitude is such a cue for UAV imagery because it shapes object size, viewing angle, and density at the same time.

To make the altitude cue usable inside a shared encoder, the tag h is mapped to a low-dimensional embedding through a multilayer perceptron, and the embedding is then transformed into channel-wise scale and shift parameters (γ, β) applied to each pyramid level (Perez et al., 2018). For a pyramid feature map F, the modulated feature F′ is obtained as shown in Equation 5:

$$F^{\prime }=\gamma (h)⊙F+\beta (h)$$ (5)

which preserves the spatial resolution of the original map while letting the network retune its response statistics for the altitude in question. The tag is encoded as an ordinal scalar, with the low, medium, and high bands mapped to 0, 0.5, and 1, so that a single two-layer perceptron of hidden width 64 generates (*γ*, *β*) for every band. The low → high protocol thus updates one shared mapping rather than three independent embeddings, and the medium-band coefficients follow by interpolation within it, which explains the gain on the intermediate band although that band receives neither supervision nor adaptation targets. Because the ordinal values feed a nonlinear two-layer perceptron rather than acting as coefficients on the features, the learned mapping from altitude to the scale and shift parameters is itself nonlinear and does not translate equal scalar spacing into equal feature displacement, and a sensitivity comparison of alternative spacings is reported in Section 3.4. This form of conditioning is more parameter-efficient than feeding altitude as an additional input channel, and it integrates naturally with the adversarial discriminator that operates on the same pyramid. Modulation is applied at all five pyramid levels, after the output convolution of the feature pyramid and ahead of the region proposal network, the detection head, and the discriminator.

The domain discriminator is a lightweight head attached to each pyramid level, formed by two 3 × 3 convolutions of 256 channels with leaky rectified activations and a 1 × 1 convolution that returns a single-channel domain map on which a per-location binary cross-entropy is evaluated; one set of weights is shared by the five levels P2 to P6, and the gradient reversal layer precedes the discriminator input so that the encoder receives the negated domain gradient. The adversarial loss is averaged across pyramid levels to balance contributions from large and small receptive fields. Combining the gradient reversal layer with feature-wise modulation gives the encoder a principled way to align cross-altitude features while keeping a representation that remains responsive to altitude-specific geometry. Treating altitude as an exploitable condition rather than as a confounder to be removed distinguishes this design from nuisance-disentanglement approaches that target invariance to altitude as one of several attributes, and it complements multi-granularity alignment strategies that operate on appearance only (Zhang et al., 2024). The gradient-reversal discriminator and the modulation act on complementary objects rather than along a single opposed axis, since the discriminator aligns the marginal source and target feature distributions to make them domain-indistinguishable while the modulation supplies a conditional altitude-specific calibration, a pairing of marginal alignment with conditioning that is standard in domain-adaptive detection rather than self-defeating. The component ablation supports this reading, because removing the adversarial discriminator lowers the high-altitude accuracy by 4.8 points and removing the modulation lowers it by 6.5 points, whereas a configuration retaining only the pseudo-label self-training reaches 36.3 mAP@0.5, so enabling both components attains 46.3 and exceeds the 45.0 that their separate gains would predict, a mild super-additivity indicating that the two objectives reinforce rather than cancel.

### 2.3 Cross-altitude pseudo-label self-training with weak-to-strong augmentation

The second stream addresses the scarcity of high-altitude annotations through a teacher-student schedule. Mean-teacher consistency regularisation has shown that an exponentially averaged teacher produces stable pseudo-labels for unlabelled samples (Tarvainen and Valpola, 2017), and follow-up work has extended this idea to object detection in both semi-supervised (Liu et al., 2021) and cross-domain (Deng et al., 2021) settings. A cross-domain variant couples a weak-to-strong augmentation contrast with adversarial alignment (Li et al., 2022), and more recent refinements include contrastive feature alignment between teacher and student and inter-class dynamics modelling tailored to object detection adaptation (Kennerley et al., 2024). The present design follows this lineage and adapts it to the cross-altitude case (Figure 2).

**Figure 2:**
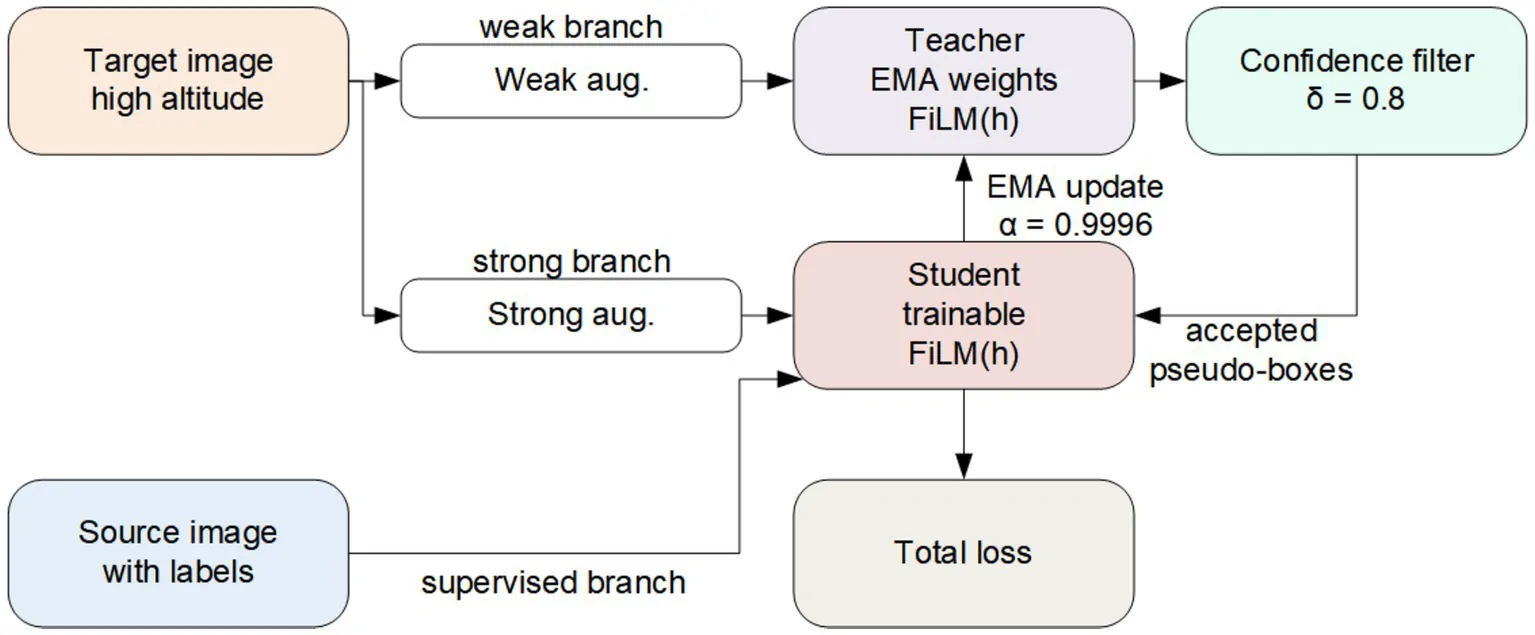
Weak-to-strong teacher-student pseudo-label workflow.

For each target image, two views are generated. A weakly augmented view, obtained by horizontal flipping and mild colour jitter, is fed to the teacher, whose predictions above a confidence threshold *δ* = 0.8 are retained as pseudo-boxes, reduced by class-agnostic non-maximum suppression at an intersection-over-union threshold of 0.5, and cleared of boxes shorter than four pixels on either side. A strongly augmented view, which inherits the flip of the weak view so that the retained boxes remain spatially valid and adds photometric distortion, Gaussian blurring, and random erasing, is fed to the student and supervised by the retained pseudo-boxes through the same detection loss used for the source. The teacher is updated through an exponential moving average of the student weights with coefficient *α* = 0.9996, while the student receives gradients from all three loss terms. A balancing schedule down-weights the unsupervised term during early iterations to prevent pseudo-label noise from dominating the optimisation before the teacher stabilises.

The same pipeline accommodates both the unsupervised setting, where no target annotation is used, and the label-efficient setting, where a small fraction of high-altitude annotations is added to the supervised loss. The altitude prior h is supplied to the teacher and the student alike, so that pseudo-labels are generated under the same feature modulation that the student is trained to use at inference. This consistency closes a loop that pure self-training pipelines rarely make explicit, and it interacts with the adversarial stream through the shared conditioned encoder rather than through the teacher directly, since the high decay coefficient of the moving average smooths the per-step adversarial fluctuation and forwards only its accumulated directional effect to the teacher, a dependence whose stability is characterised by the decay-coefficient sensitivity in Table 1. The full training procedure is summarised in Algorithm 1.

Algorithm 1 Training workflow for CADA-Net.
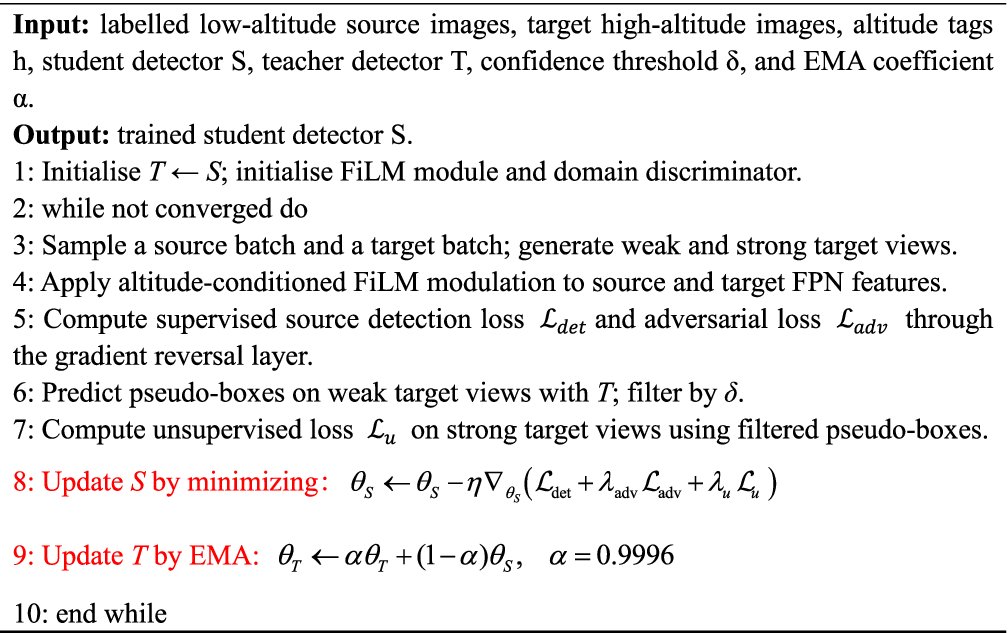

**Table 1:** Sensitivity analysis of the EMA decay coefficient.

EMA decay *α*	High mAP@0.5	Overall mAP@0.5
0.9990	45.5	58.5
0.9996 (default)	46.3	58.9
0.9999	46.1	58.8

Values are mAP@0.5. High denotes the high-altitude band and overall is computed over the pooled test set. The coefficient *α* = 0.9996 is the default used in all other experiments.

### 2.4 Datasets and cross-altitude adaptation protocol

UAVDT serves as the principal benchmark because it ships with altitude metadata at the sequence level, which propagates to every frame within the sequence and places each frame in a low, medium, or high band (Du et al., 2018). The detection subset used here comprises about 40,000 annotated frames drawn from the roughly 80,000-frame UAVDT benchmark, with vehicle traffic recorded under varied weather and viewpoints. The three vehicle subclasses in the original annotation are merged into a single vehicle class to align the evaluation with earlier UAV robust-detection work that adopts the same convention. Cross-altitude transfer is studied along the principal low → high protocol, in which low-altitude frames provide the labelled source and high-altitude frames provide the target. Partitioning follows the official UAVDT detection protocol at the level of complete video sequences, so that every frame of a sequence falls on one side of the split and no sequence contributes frames to both, which prevents near-duplicate frames from a single continuous shot from appearing in training and test alike. The sequence lists of each band and partition are released with the split files.

Table 2 summarises the UAVDT split sizes, box counts, altitude distributions, and resolutions used in the study. The high-altitude training subset contains only 352 frames, which mirrors the practical scarcity of high-altitude annotations encountered during real-world UAV deployment. The high-altitude frames originate from a small number of sequences, so box density varies notably between the training and test partitions. Medium-altitude frames are used neither as the labelled source nor as the adaptation target under this protocol; they are held out and used only to evaluate how the adapted detector behaves on an intermediate band.

**Table 2:** Dataset statistics and altitude distribution.

Split	Images	Boxes	Altitude distribution	Categories	Resolution/note
UAVDT train	24,143	422,911	high: 352; low: 7,657; medium: 16,134	vehicle	1,080 × 540
UAVDT test	16,592	375,884	high: 1,680; low: 6,987; medium: 7,925	vehicle	1,080 × 540
Low source	7,657	139,238	low: 7,657	vehicle	1,080 × 540
High target train	352	4,583	high: 352	vehicle	1,080 × 540
High target test	1,680	104,466	high: 1,680	vehicle	1,080 × 540
Medium intermediate	16,134	279,090	medium: 16,134	vehicle	1,080 × 540

### 2.5 Implementation details and evaluation metrics

CADA-Net is implemented within the MMDetection framework on PyTorch. The backbone is initialised from ImageNet pretrained ResNet-101 weights, and the FPN neck produces multi-scale feature maps for the detector and domain discriminator. The reference RPN uses anchors with scale 8, aspect ratios 0.5, 1.0, and 2.0, and strides of 4, 8, 16, 32, and 64 pixels. Images are resized to a working resolution of 1,024 × 540 prior to training and inference, from the original UAVDT resolution of 1,080 × 540; the training batch size is 4 and the validation/test batch size is 1. Training is carried out on a single NVIDIA RTX 3090 GPU, while onboard latency is profiled separately on the Jetson AGX Orin described below. All reported values originate from a single training run under a fixed random seed, since training the full pipeline on the cross-altitude protocol exhausts the available computational budget, and the comparison is therefore presented as an ordering of methods under a common protocol rather than as a statistically tested difference. Optimisation uses stochastic gradient descent with momentum 0.9, weight decay 0.0001, and an initial learning rate of 0.005 decayed by a cosine schedule over 30 epochs. The exponential moving average coefficient for the teacher is 0.9996 and the pseudo-box confidence threshold δ is 0.8, following values reported as stable in earlier teacher-student detection work (Li et al., 2022). The adversarial loss weight $\lambda_{\mathrm{adv}}$ is held at 0.1 throughout, the unsupervised loss weight λu rises linearly from 0 to 1 over the first three epochs and remains at unity thereafter, and the FiLM generator has a hidden width of 64.

Detection accuracy is measured by mAP@0.5 and mAP@0.5:0.95. mAP@0.5 is reported per altitude band and over the pooled test set, while mAP@0.5:0.95 is reported over the pooled test set. Onboard efficiency is profiled on an NVIDIA Jetson AGX Orin in 60 W MAXN mode, with model parameters in millions, end-to-end latency in milliseconds, frames per second, and average power draw recorded for each runtime format. Two comparison families are considered: domain-adaptive detectors that share the ResNet-101/FPN backbone, and modern real-time detectors that represent no-adaptation single-domain practice (Zhao et al., 2024; Wang et al., 2024; Hussain, 2024).

## 3 Results

### 3.1 Comparison with state-of-the-art methods

The principal comparison evaluates CADA-Net against seven competing approaches on the low → high adaptation protocol. The set covers a source-only lower bound, a fully-supervised reference trained with annotations across all altitude bands as a strong upper bound for the cross-altitude evaluation, two canonical domain-adaptive detectors built on adversarial alignment (Chen et al., 2018) and on teacher-student self-training, a UAV-specific nuisance-disentanglement baseline (Wu et al., 2019), and two modern real-time detectors evaluated as no-adaptation baselines. Per-altitude mean average precision, model size, and inference speed are reported in Table 3. Every competing entry was retrained rather than transcribed from its original publication, using the officially released implementation under the ResNet-101 backbone where the method admits one, the same input resolution and 30-epoch schedule, and the split of Section 2.4, with throughput measured for all entries on the same Jetson AGX Orin platform, so that the reported differences follow from the adaptation mechanism rather than from divergent training budgets or evaluation hardware. More recent teacher-student methods such as the contrastive mean teacher (Cao et al., 2023) and the inter-class dynamics framework (Kennerley et al., 2024) share the lineage of the present design, yet both derive their supervisory signal from contrasts between object categories, and these degenerate once the three UAVDT vehicle subclasses are merged into one vehicle class, so tabulating them here would measure a disabled mechanism rather than the method itself.

**Table 3:** Comparison with state-of-the-art methods on UAVDT (low → high).

Method	Backbone	Low mAP@0.5	Med mAP@0.5	High mAP@0.5	Overall mAP@0.5	mAP@0.5:0.95	Params (*M*)	FPS	High gain
Source-only	ResNet-101/FPN	63.8	51.2	28.4	49.7	25.1	41.5	18.4	0.0
Fully-supervised reference	ResNet-101/FPN	66.1	60.8	52.6	60.4	35.8	41.5	18.4	+24.2
DAF	ResNet-101/FPN	63.1	53.5	33.2	52.2	27.8	42.0	17.9	+4.8
Adaptive teacher	ResNet-101/FPN	64.4	55.8	39.1	56.1	30.7	42.8	16.7	+10.7
NDFT	ResNet-101/FPN	64.0	54.7	36.4	54.4	29.5	44.2	15.9	+8.0
YOLOv10 (no adaptation)	YOLOv10-S	59.8	50.2	31.7	50.6	26.3	15.8	54.0	+3.3
RT-DETR (no adaptation)	R50	62.4	53.1	35.6	53.4	28.9	42.0	25.0	+7.2
CADA-Net (this work)	ResNet-101/FPN	65.2	58.7	46.3	58.9	33.9	44.7	16.2	+17.9

Fully-supervised reference denotes a detector trained with annotations across all altitude bands and serves as a strong upper bound for the cross-altitude protocol. Overall mAP is computed over the pooled test set rather than as an average of per-band values. mAP@0.5:0.95 is pooled. All FPS values are measured on the same NVIDIA Jetson AGX Orin platform using PyTorch FP32 at 1024 × 540 input for a fair head-to-head comparison. YOLOv10 and RT-DETR are no-adaptation single-domain baselines. Values are reported from the single reference run, and no claim of statistical significance is made.

CADA-Net reaches 46.3 mAP@0.5 on the high-altitude band, which corresponds to a gain of 17.9 points over the source-only baseline and 7.2 points over the strongest domain-adaptive competitor. The gap to the fully supervised upper bound narrows to 6.3 points, a margin that is worth noting given that the high-altitude training set contains only 352 frames. This figure refers to the target adaptation partition rather than to the data available to either model, since the detector draws its supervised signal from the 7,657 labelled low-altitude frames on an ImageNet-pretrained ResNet-101 backbone while the 352 high-altitude frames serve as unlabelled adaptation targets under the principal protocol, and the fully-supervised reference is trained with labels across the full 24,143-frame partition, so the residual gap measures the difficulty of cross-altitude transfer rather than a shortage of data in the upper bound; the label-efficient analysis in Section 3.3 corroborates this, since supplying all 352 high-altitude labels raises the model to 51.0 mAP@0.5 and reduces the distance to the reference to 1.6 points. Performance on the low- and medium-altitude bands also improves, which suggests that altitude-conditioned modulation acts as a regulariser across bands rather than trading source accuracy for target accuracy. Real-time single-domain detectors remain competitive in latency but trail CADA-Net by about 11 to 15 points and trail the Adaptive Teacher by about 4 to 7 points on the high-altitude band, while remaining comparable to weaker adaptation baselines such as DAF and NDFT. This pattern echoes recent observations that modern detector backbones do not, on their own, resolve domain shift (Oza et al., 2023).

The training dynamics in Figure 3 indicate that CADA-Net attains a higher plateau and also continues to improve past the iteration count at which the adversarial-only baseline has saturated. The teacher-student baseline climbs further than the adversarial baseline, which is consistent with the pseudo-label-driven adaptation literature, but it plateaus around 39 mAP@0.5 on the target band, several points below the proposed method. The additional headroom traces back to the altitude-conditioned modulation. By supplying both the teacher and the student with a representation already tuned to the target geometry, the modulation reduces the amount of appearance noise carried by the pseudo-labels, which in turn lowers the variance of the consistency signal that drives the student.

**Figure 3:**
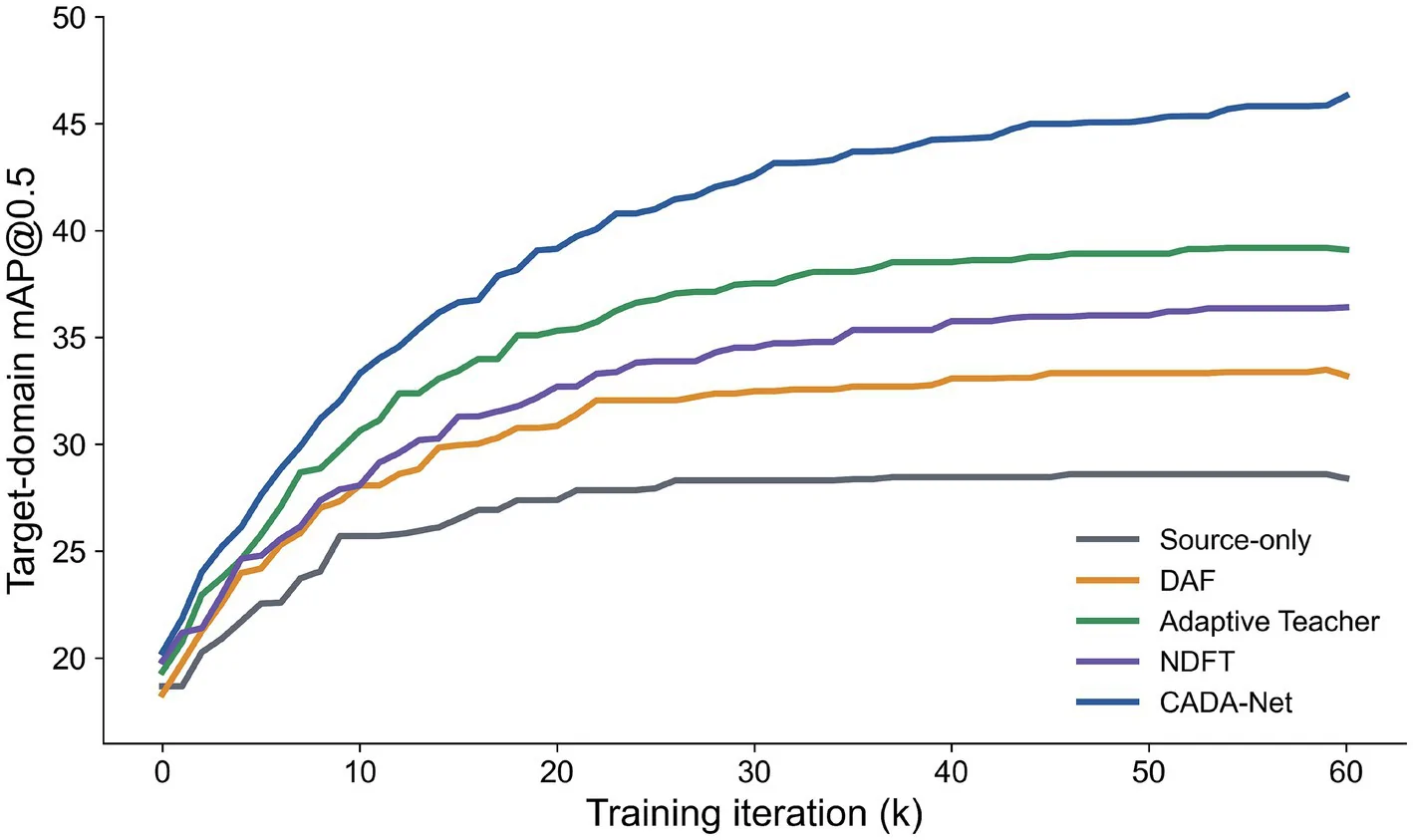
Training convergence curves on UAVDT cross-altitude adaptation.

### 3.2 Is cross-altitude shift a scale problem? A controlled analysis

A natural objection to altitude-conditioned adaptation is that altitude variation could simply be a proxy for object scale variation. If that were the case, multi-scale training and feature pyramids alone would close the gap. This hypothesis is examined directly from two angles in Figures 4a, b.

**Figure 4:**
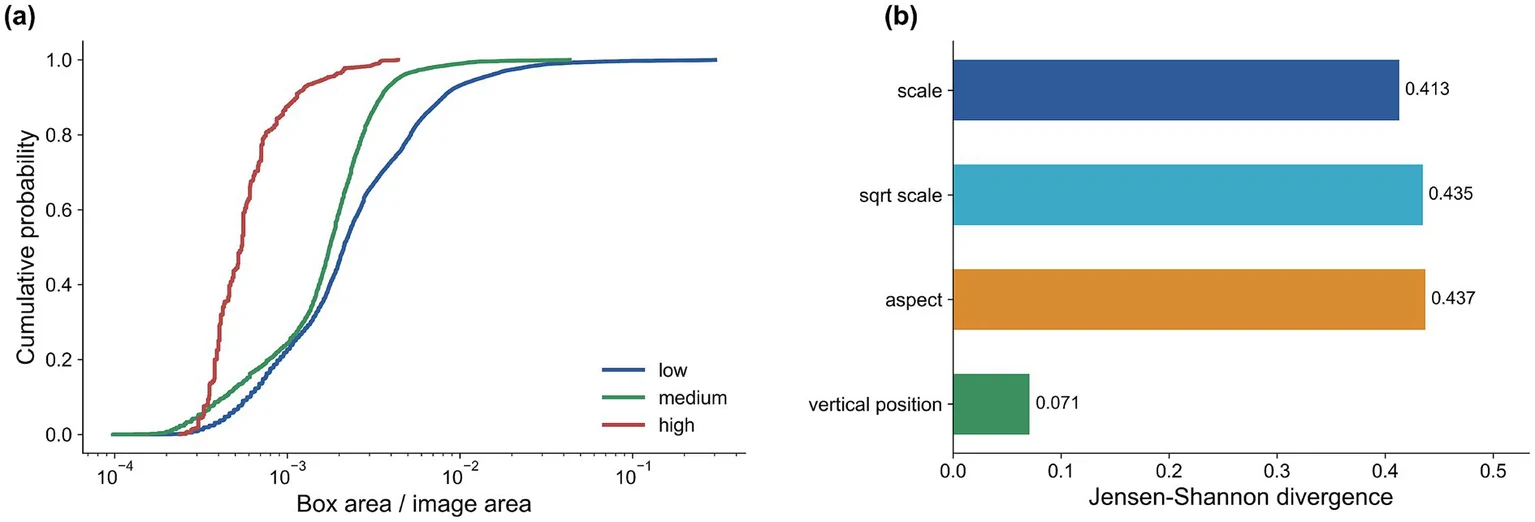
Cross-altitude shift is not reducible to scale variation. **(a)** Cumulative distribution of normalised box area (box area/image area) for the low-, medium-, and high-altitude bands. **(b)** Jensen-Shannon divergence between the low- and high-altitude marginal distributions of four image-level statistics (scale, square-root scale, aspect ratio, and vertical position).

The cumulative distribution of normalised box area in panel (a) shows that high-altitude objects are visibly smaller than low- and medium-altitude objects, but the three distributions overlap substantially between 10^−3^ and 10^−2^ of normalised box area. Scale alone therefore does not provide a clean separation. To make this observation quantitative, panel (b) reports the Jensen-Shannon divergence between the low- and high-altitude marginal distributions on four image-level statistics. Box scale and its square root yield divergences of 0.413 and 0.435, aspect ratio reaches 0.437, and vertical position remains low at 0.071. Aspect ratio carries a divergence comparable to scale, which indicates that the cross-altitude shift involves a viewpoint component that scale-augmentation strategies cannot reach.

A complementary line of evidence comes from comparing scale-only and altitude-aware training under matched protocols. A multi-scale baseline that augments across object sizes but ignores altitude leaves a substantial residual gap on the high-altitude band, which altitude conditioning closes. The pattern is informative on two counts. The residual gap left by scale augmentation shows that multi-scale capacity does not absorb the geometric and semantic component of the shift, and the close fit between altitude-aware variants with and without additional multi-scale augmentation suggests that the modulation already exposes the encoder to a sufficient range of effective receptive fields, which makes additional multi-scale augmentation partially redundant.

Taken together, the evidence shows that the cross-altitude gap is geometric and semantic at the same time, in line with recent surveys that group geometric shifts together with semantic shifts in the domain adaptation literature. The implication is practical. A UAV detector intended for multi-altitude flight benefits from making altitude a first-class input rather than relying on scale-invariant features alone, even when the backbone is already a pyramidal one.

### 3.3 Label-efficient adaptation under varying annotation budgets

The label-efficient evaluation varies the target-domain annotation budget across 0, 1, 5, 10, and 100% of the 352 available high-altitude training frames, which amounts to 4, 17, and 35 annotated frames at the three intermediate settings, with CADA-Net and the adaptive teacher baseline trained under each budget. The annotated frames are drawn by random sampling stratified over the high-altitude sequences under a fixed seed, so that no single sequence dominates the smallest budgets, and the selections are nested, each smaller budget being contained in the next. Identical frame lists are supplied to every method compared at a given budget, which removes subset selection as a source of the differences between the curves. The mAP@0.5 on the high-altitude test set is reported in Figure 5, with the source-only baseline and the fully-supervised upper bound shown as references.

**Figure 5:**
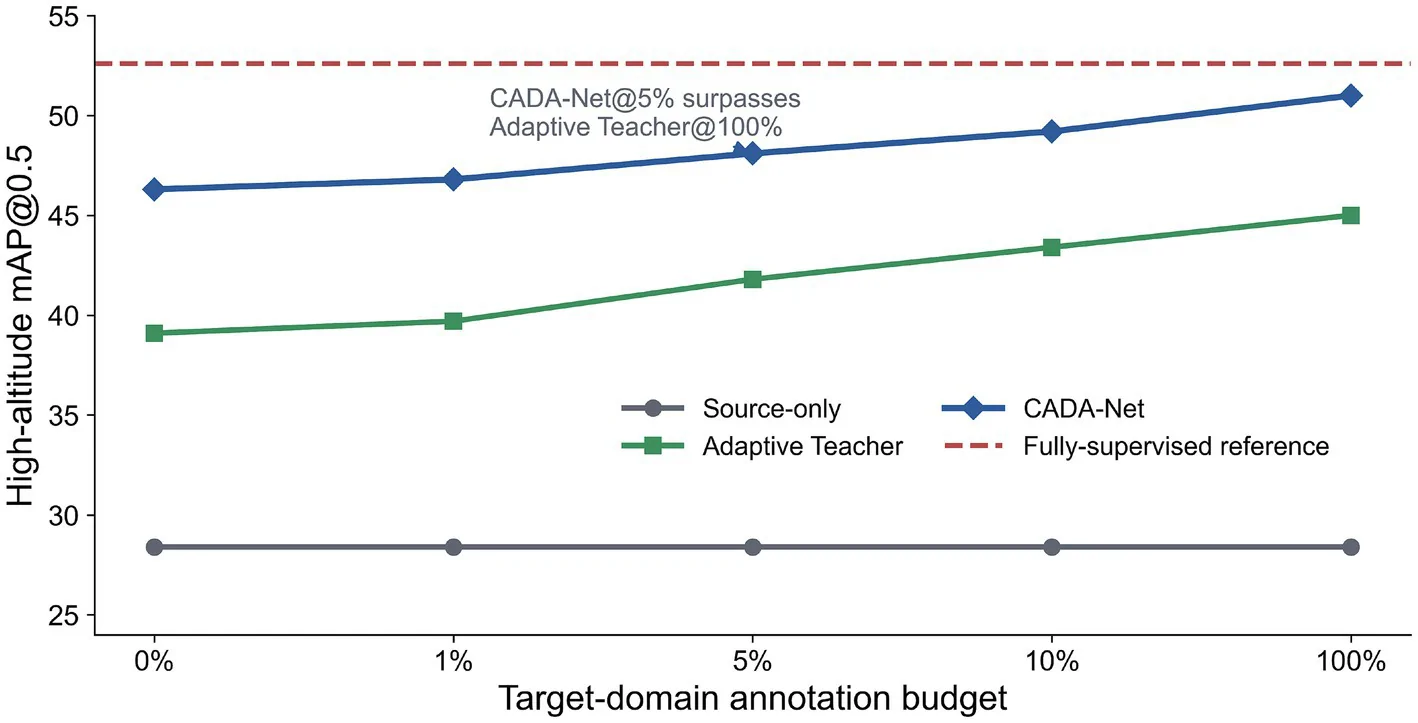
Label efficiency under varying target-domain annotation budgets.

CADA-Net at 0% target labels already reaches 46.3 mAP@0.5, which exceeds the adaptive teacher baseline even when the latter is trained with all 352 high-altitude labels (45.0 mAP@0.5; see Figure 5). At 5% of the target labels (17 high-altitude frames), CADA-Net further improves to 48.1 mAP@0.5, widening this margin. At 100% target labels, CADA-Net reaches 51.0 mAP@0.5 and approaches the fully-supervised upper bound to within 1.6 points. The shape of the curve is roughly logarithmic, with most of the improvement secured by the time the budget reaches 5%. This pattern is consistent with theoretical expectations for self-training under bounded pseudo-label noise (Munir et al., 2023).

The comparison to the adaptive teacher baseline is informative beyond the aggregate gap. The adaptive teacher curve climbs more steeply between 10 and 100% because the underlying detector lacks altitude conditioning, so additional target labels mainly help the network resolve appearance ambiguities. The CADA-Net curve is flatter because altitude-conditioned modulation already absorbs a large part of the geometric shift, so the marginal value of each additional label diminishes earlier. From the perspective of mission-time annotation budgets in UAV deployment, this pattern is helpful. A small number of labels at a new altitude is sufficient to recover most of the available accuracy, which lowers the cost of retraining the perception module after operational changes.

The label-efficient profile also speaks to the earlier scale-versus-altitude question. If the shift were purely a scale problem, the source-only baseline would benefit from additional target labels, but the source-only line remains essentially flat because no annotation budget changes the underlying encoder. Only altitude-aware self-training and adversarial alignment narrow the gap, which reinforces the view that altitude is an explanatory variable that earlier UAV detectors have left underused.

### 3.4 Ablation study on key components

The ablation in Table 4 isolates the contribution of each component of CADA-Net. Removing FiLM modulation reduces the high-altitude mAP by 6.5 points, removing the adversarial discriminator reduces it by 4.8 points, and disabling pseudo-label self-training causes the largest single drop at 9.1 points. The relative magnitudes match the design intent. Pseudo-label self-training drives the bulk of the cross-altitude transfer when target annotations are scarce, while altitude-conditioned modulation supplies the geometric prior that keeps the pseudo-labels reliable. The adversarial term contributes a smaller but consistent share by smoothing the feature distribution across altitudes.

**Table 4:** Ablation study on key components and conditioning strategies.

Config	Adv.	Altitude cond.	Pseudo-label	Low	Med	High	Overall	High gain
Full CADA-Net	✓	FiLM	✓	65.2	58.7	46.3	58.9	+17.9
w/o FiLM	✓	–	✓	64.1	55.3	39.8	55.1	+11.4
w/o adversarial	–	FiLM	✓	64.4	56.2	41.5	56.2	+13.1
w/o pseudo-label	✓	FiLM	–	63.9	54.8	37.2	54.0	+8.8
Concat conditioning	✓	concat	✓	64.6	56.8	42.7	56.9	+14.3
Embedding branch	✓	embedding	✓	64.8	57.1	43.5	57.4	+15.1
Multi-scale baseline	–	multi-scale	–	64.5	54.2	34.6	53.6	+6.2
Multi-scale + CADA	✓	FiLM + multi-scale	✓	65.0	58.2	45.2	58.3	+16.8

Ablations follow the same low → high protocol as Table 3. Values are reported from the single reference run, and no claim of statistical significance is made.

The choice of conditioning injection also matters. Replacing FiLM modulation with feature concatenation lowers the high-altitude mAP by 3.6 points, and a separate embedding branch trails the full configuration by 2.8 points. The result is consistent with the original observation that channel-wise affine modulation is a parameter-efficient way to inject categorical conditioning into convolutional features (Perez et al., 2018), and it extends that observation to a detection setting in which the condition is a metadata tag rather than a language token. The multi-scale baseline and the multi-scale-plus-CADA configurations sit at the bottom and the second-highest position of the table, with the latter matching the full model to within 1.1 points.

Whether the uniform ordinal spacing matches the nonlinear change of viewpoint across bands is examined by replacing the linear encoding with a square-root spacing that widens the sharper low-to-medium interval and with a fully learnable scalar per band, under an otherwise identical protocol, as reported in Table 5.

**Table 5:** Sensitivity analysis of altitude encoding strategies.

Altitude cond.	Encoding	Low	Med	High	Overall
None	–	64.1	55.3	39.8	55.1
FiLM	Linear	65.2	58.7	46.3	58.9
FiLM	Square-root	65.2	59.3	46.6	59.2
FiLM	Learnable	65.1	58.5	45.8	58.6

Values are mAP@0.5. Low, Med, and High denote the low-, medium-, and high-altitude bands, and overall is computed over the pooled test set. The none and linear rows coincide with the w/o-FiLM and full CADA-Net configurations in Table 4.

The square-root spacing, which widens the interval assigned to the sharper low-to-medium transition, raises the medium band from 58.7 to 59.3 mAP@0.5 and leaves the high band effectively unchanged at 46.6 against 46.3, whereas the learnable scalar trails the linear encoding by 0.5 points on the high band, consistent with the difficulty of fitting free band positions from only two supervised endpoints under the 352-frame budget. Linear spacing is therefore a robust default rather than a strict optimum, the benefit of a nonlinear reshaping falls almost entirely on the held-out medium band that the low → high protocol never supervises, and the headline high-altitude gain is insensitive to the exact spacing, so the geometric prior rather than the particular scalar coordinates carries the improvement.

The dependence of the teacher on the smoothing strength of its update, which sets how much of the adversarially driven drift in the student is retained rather than filtered, is examined by varying the exponential-moving-average coefficient around its default, with the outcome reported in Table 1.

Reducing the coefficient to 0.9990 lowers the high-altitude accuracy to 45.5 mAP@0.5, since a faster-tracking teacher inherits more of the per-step fluctuation and yields noisier pseudo-labels, while raising it to 0.9999 slightly reduces the accuracy to 46.1 as the teacher responds too slowly to the aligned representation, so the default 0.9996 sits at the balance between the two. The narrow 0.8-point spread indicates a coupling that is stable rather than fragile, and the observation that neither a weaker nor a stronger filter improves on the default is consistent with a moving average that forwards the accumulated directional effect of the adversarial stream to the teacher while attenuating its transient fluctuation.

### 3.5 Qualitative visualization and onboard deployment analysis

Qualitative comparisons in Figure 6 illustrate detector behaviour across the three altitude bands. At the low and medium altitudes, CADA-Net produces tighter bounding boxes than the source-only baseline on partially occluded vehicles, and it suppresses several false positives produced by the adaptive teacher baseline on cluttered intersections. At the high altitude, where targets are small and the background is dominated by haze, the three methods retrieve a similar number of objects, but the CADA-Net boxes align more closely with vehicle silhouettes.

**Figure 6:**
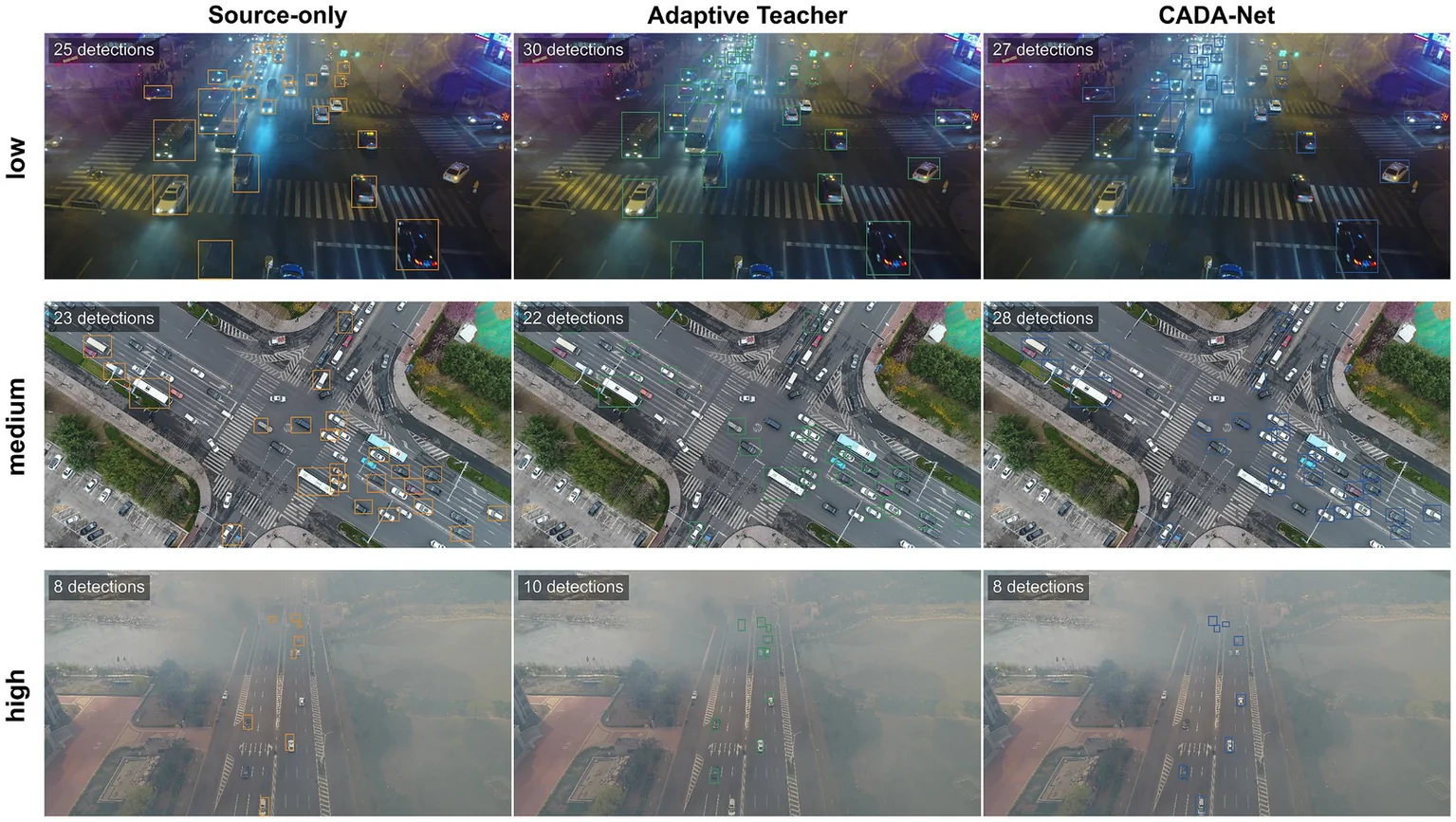
Qualitative detection comparison across altitudes. Source frames from the UAVDT benchmark (Du et al., 2018), used under non-commercial academic terms.

The visual contribution of the model in this regime therefore lies in localisation accuracy rather than in raw object count. Feature-space evidence in Figure 7 provides the corresponding mechanistic view. Before adaptation (Figure 7a), t-SNE embeddings separate cleanly by altitude band, with red, green, and blue clusters occupying distinct regions of the projection. After CADA-Net adaptation (Figure 7b), the three colours interleave while still preserving a coarse semantic ordering, a pattern expected from a successful alignment that retains class-relevant geometry.

**Figure 7:**
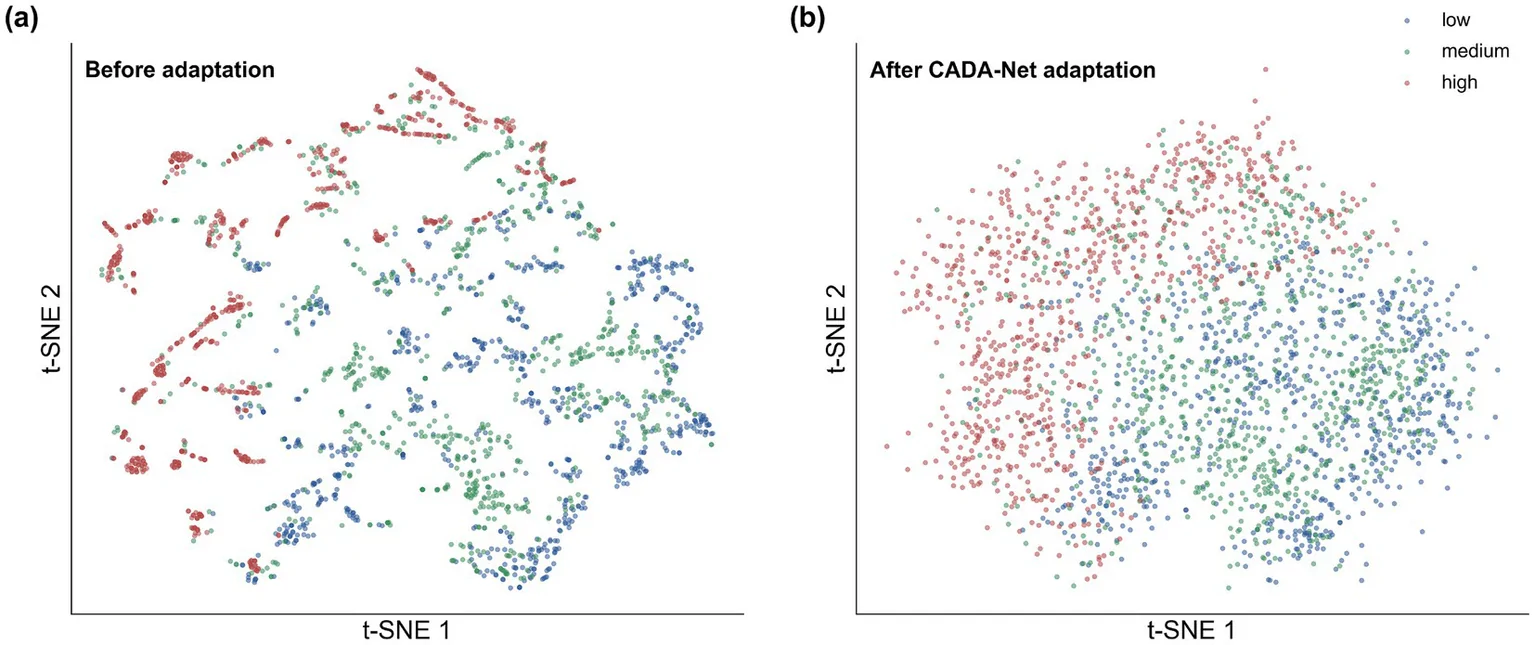
Feature alignment via t-SNE before and after adaptation. **(a)** Embeddings before adaptation. **(b)** Embeddings after CADA-Net adaptation. Points are coloured by altitude band as indicated in the legend.

The onboard profile in Table 6 indicates that the accuracy gain is retained under embedded inference. The PyTorch FP32 reference reaches 16.2 frames per second on a Jetson AGX Orin at 1024 × 540 input, ONNX FP32 increases to 20.7, and TensorRT FP16 reaches 32.3 with a 0.5-point accuracy drop. The TensorRT INT8 build attains 42.0 frames per second with a 1.4-point accuracy drop and an average power draw of 27.4 W, which leaves substantial headroom within the 60 W envelope of the platform. Figure 8 places CADA-Net on an accuracy-efficiency frontier alongside the comparison methods. CADA-Net leads on high-altitude mAP@0.5 at a parameter budget comparable to the other adaptation baselines; its FP32 throughput is lower than that of the single-domain real-time detectors, while INT8 quantisation restores real-time operation at 42 frames per second (Table 6). The profiling exercises the perception module alone, so the figures bound onboard feasibility rather than demonstrate closed-loop operation.

**Table 6:** Onboard deployment efficiency on Jetson AGX Orin.

Runtime format	mAP@0.5	Latency (ms)	FPS	Accuracy loss	Power (*W*)
PyTorch FP32	46.3	61.8	16.2	0.0	42.5
ONNX FP32	46.1	48.4	20.7	−0.2	38.0
TensorRT FP16	45.8	31.0	32.3	−0.5	31.6
TensorRT INT8	44.9	23.8	42.0	−1.4	27.4

Measured on NVIDIA Jetson AGX Orin in 60 W MAXN mode. Input resolution: 1024 × 540. Accuracy loss is reported relative to the PyTorch FP32 baseline.

**Figure 8:**
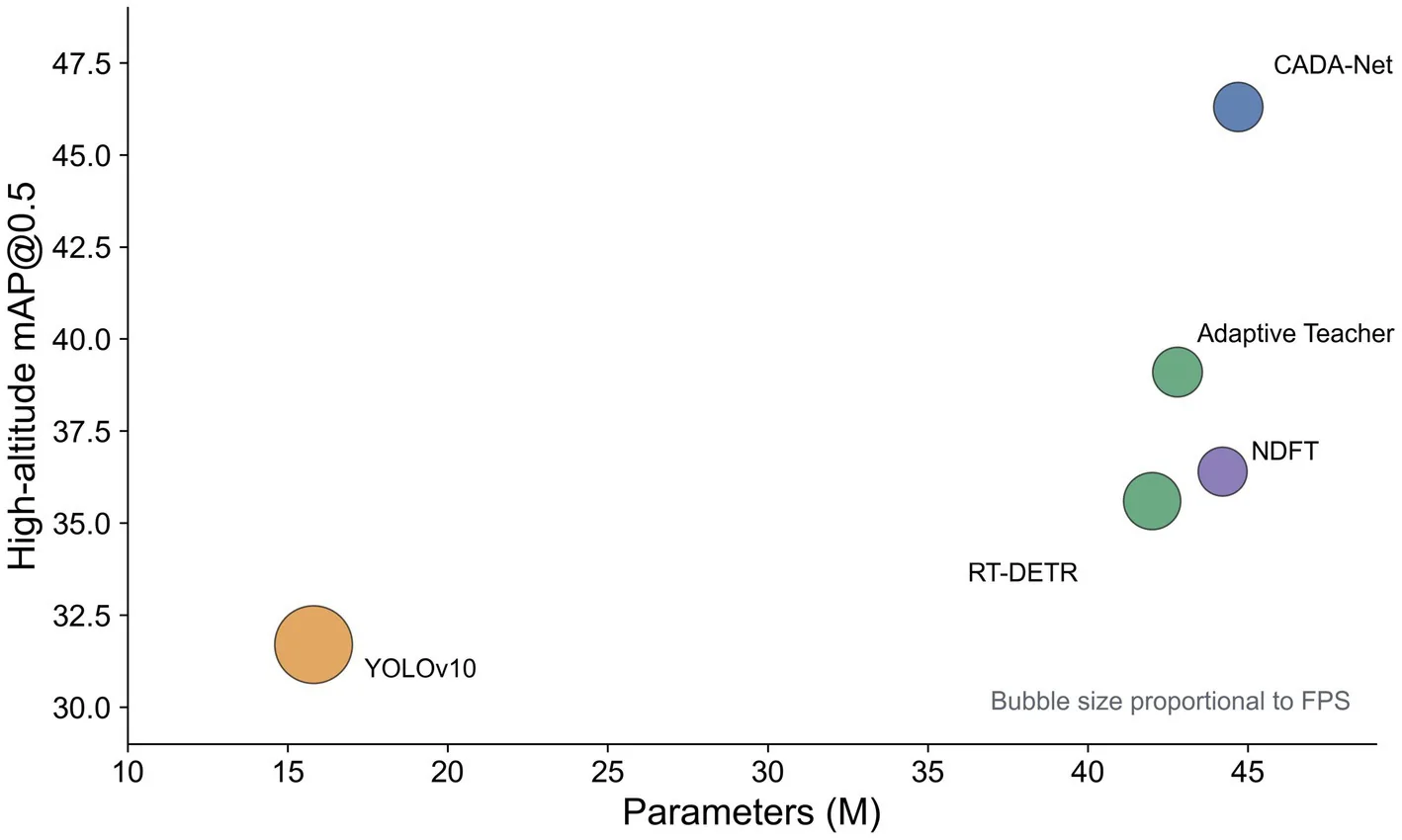
Accuracy-efficiency Pareto frontier on UAVDT.

## 4 Discussion

The cross-altitude transfer gap that CADA-Net narrows is best read as a coupled geometric and semantic shift rather than as a single appearance shift. The image-level divergence analysis makes this concrete: aspect ratio carries roughly as much information as scale about the band membership of an image. A detector that aligns only the appearance distribution, in the manner of methods originally designed for weather and style transfer, therefore leaves a structural residual untreated. The same reasoning helps to explain why approaches that remove altitude as one of several confounders underperform conditional approaches on this benchmark. Removing altitude discards information that the encoder needs to retune its receptive field, while exposing altitude as a modulation signal lets the encoder use that information without committing the discriminator to a single target manifold. The fact that channel-wise modulation outperforms concatenation and a separate embedding branch in Table 4 echoes the broader finding that feature-wise affine modulation is an effective way to inject categorical conditioning into convolutional features, and the present results suggest that this finding carries over to UAV detection settings where the condition is a piece of platform metadata.

The relation of CADA-Net to teacher-student baselines deserves a closer look. Pseudo-label-driven adaptation has become a dominant strategy in cross-domain detection, in part because it sidesteps the brittleness of pure adversarial training (Kennerley et al., 2024). The component ablation reported in this work shows that pseudo-labels still provide the largest single contribution to the cross-altitude gain, yet pseudo-labels alone are not enough because the teacher itself benefits from altitude conditioning when it generates supervisory signals. The pairing of conditioning and self-training therefore behaves less like a sum of independent contributions and more like a feedback loop in which each component improves the other. This view is in line with recent multi-granularity alignment frameworks that interleave coarse and fine alignment signals (Zhang et al., 2024), and it adds a small piece of evidence to the broader case that domain adaptation for detection benefits from informative priors rather than from invariance for its own sake.

Several limitations should be acknowledged. The altitude tag used in this work is a three-band discretisation rather than a continuous reading, which works on UAVDT but may understate the gain on platforms whose altimeters return finer-grained readings; the sensitivity comparison in Table 5 shows that a square-root spacing marginally improves the held-out medium band while leaving the high band unchanged, so the linear encoding is retained as a robust default and a continuous altitude embedding is left as the natural extension. The pseudo-label filter is governed by a single confidence threshold, and dynamic targets at high altitude occasionally generate noisy pseudo-boxes that the current schedule does not fully suppress. Evaluation is confined to UAVDT, which remains the only public UAV detection benchmark whose sequences carry the altitude attribute that the conditioning requires as its domain variable, so confirming cross-dataset generality once a second altitude-annotated benchmark becomes available is left to future work. All reported numerical results come from a single training run, and the relatively small variance typical of this benchmark under the reference configuration has not been quantified through multi-seed repetition. A repetition of the principal configurations under several random seeds, with the mean and standard deviation reported for each, is the appropriate way to convert the present ordering into a statistically supported claim and is planned for the extended version of this work. The settings of δ, $\lambda_{\mathrm{adv}}$ , and the EMA coefficient α follow stable values from teacher-student and adversarial detection baselines, but a broader hyperparameter sensitivity sweep remains necessary before deployment in a new flight regime. Real-platform deployment further introduces motion blur, exposure variation, and communication latencies that an offline Jetson profile does not capture. In downstream autonomy, these detector errors would not be neutral: missed vehicles can delay avoidance decisions, whereas false positives can cause unnecessary waypoint changes. Future work should therefore embed CADA-Net in closed-loop UAV navigation and avoidance pipelines, following the broader embodied-perception direction represented by NavBLIP (Li et al., 2025), to evaluate task-level outcomes such as collision rate, unnecessary avoidance manoeuvres, and waypoint tracking error rather than detection accuracy in isolation.

## 5 Conclusion

Across these experiments, the three hypotheses are borne out. Making altitude an explicit, FiLM-injected domain variable recovers more of the gap than altitude-agnostic alignment (H1); the teacher-student stream sustains competitive accuracy with few or no target labels (H2); and a strong multi-scale baseline alone leaves a residual gap that altitude-aware adaptation narrows (H3). This study has presented CADA-Net, a cross-altitude domain-adaptive detector that treats altitude as an explicit domain variable and combines altitude-conditioned feature modulation, gradient-reversal adversarial alignment, and weak-to-strong teacher-student self-training inside a shared encoder. Evaluation on UAVDT shows a 17.9-point gain in high-altitude mean average precision over the source-only baseline and a 7.2-point gain over the strongest domain-adaptive competitor, while the gap to a fully supervised upper bound narrows to 6.3 points without using high-altitude annotations in the unsupervised setting. The label-efficient analysis suggests that around 5% of high-altitude labels is enough to surpass a fully supervised adaptive teacher baseline, and onboard profiling on Jetson AGX Orin returns 42 frames per second at INT8 precision within a 27.4 W power envelope, which establishes feasibility rather than closed-loop validation. The results offer modest evidence that an explicit, low-cost geometric prior can be a useful design ingredient for onboard perception modules in autonomous UAV platforms.

Several directions remain open. The altitude prior could be generalised from a discrete band to a continuous reading, the pseudo-label schedule could be made more adaptive to scene dynamics, and the perception backbone could be coupled with downstream navigation and avoidance controllers to evaluate task-level outcomes rather than detection metrics in isolation. Pursuing these directions should help move cross-altitude detection from a benchmark observation toward a more thoroughly validated component of autonomous UAV systems.

## Data Availability

Publicly available datasets were analyzed in this study. The UAVDT benchmark is publicly available at https://sites.google.com/view/grli-uavdt and is distributed for non-commercial academic use. No new data were generated in this study. The processed COCO-format splits, the sequence lists of each altitude band and partition, and the frame lists used for each annotation budget are archived at https://doi.org/10.5281/zenodo.21866553. The model implementation and trained checkpoints are available from the corresponding author on reasonable request.
